# Safety and Efficacy of Pamidronate in Neonatal Hypercalcemia Caused by Subcutaneous Fat Necrosis: A Case Report

**DOI:** 10.3389/fped.2022.845424

**Published:** 2022-04-28

**Authors:** Stefano Martinelli, Marco Pitea, Italo Francesco Gatelli, Tara Raouf, Graziano Barera, Ottavio Vitelli

**Affiliations:** ^1^Neonatal Intensive Care Unit, ASST Grande Ospedale Metropolitano Niguarda, Milan, Italy; ^2^Pediatric and Neonatal Unit, IRCCS San Raffaele Hospital, Milan, Italy; ^3^Vita-Salute San Raffaele University, Milan, Italy

**Keywords:** pamidronate, biphosphonate, subcutaneous fat necrosis, hypercalcemia, newborn

## Abstract

Subcutaneous fat necrosis of the newborn (SCFN) is a panniculitis that develops in fatty areas after fetal or perinatal distress. Prognosis is generally good with complete regression, but it can be complicated by metabolic abnormalities like hypoglycemia, hypertriglyceridemia, thrombocytopenia, and also potentially life-threatening hypercalcemia. Treatments have included hydration, furosemide and corticosteroids. These treatments can be prolonged for several days and can have complications such as nephrocalcinosis. Use of bisphosphonates has been rarely reported in newborn. We describe a case of severe hypercalcemia complicating subcutaneous fat necrosis in a newborn successfully treated by a single dose of pamidronate after having obtained partial response by therapy with hyperhydration, furosemide and hydrocortisone. When high levels of calcium do not respond to first line therapy with hyperhydration and diuretic therapy, bisphosphonates treatment could be considered a valid choice to treat hypercalcemia and to avoid corticosteroids. Further studies are needed to understand if pamidronate and other bisphosphonates can be considered the first choice in hypercalcemia due to SCFN.

## Introduction

Subcutaneous fat necrosis (SCFN) is a recognized but uncommon disorder characterized by firm, red or purple subcutaneous nodules and plaques on the trunk, buttocks, cheeks, and extremities, and it is associated with perinatal stress (1).

Although the pathogenesis of the disorder is currently unknown, it is most commonly observed in association with whole-body hypothermia treatment for perinatal asphyxia (2, 3). Previously described predisposing factors include localized skin trauma, obstetric trauma, preeclampsia, meconium aspiration, sepsis, maternal diabetes and decreased renal clearance of calcium (4–7).

The composition of neonatal fat has also been suggested as a possible cause, as neonatal fat has a higher melting point than adult fat due in part to a high percentage of saturated fatty acids, that leads to a predisposition for solidification and crystallization of the adipose tissue with hypothermia (8).

Hypothermia is now the standard of care in the management of newborns with moderate to severe hypoxic-ischemic encephalopathy in infants meeting the inclusion criteria (9). Adverse effects from hypothermia have been reported, however, the benefits of cooling therapy outweigh the risks. Whether SCFN is due to hypoxic-ischemia, hypothermia, or a combination of factors in infants with hypoxic-ischemic encephalopathy, which requires further evaluation.

Subcutaneous fat necrosis is usually a benign condition, however several complications can occur such as local tissue breakdown, hypoglycemia, anemia, thrombocytopenia, hypertriglyceridemia, and hypercalcemia. Hypercalcemia due to SCFN occurs in approximately 50% of neonates presenting with this condition in the first month of life and it is less frequent at an older age.

While often asymptomatic, hypercalcemia can lead to: irritability, vomiting, polyuria, failure to thrive, neurologic symptoms/seizures, hypertension, intellectual disability, renal failure, and even death (10). Treatment of hypercalcemia in SCFN is controversial and includes hyperhydration, diuretic therapy, steroids in addition to formula low in calcium and subcutaneous calcitonin as an immediate short-term management of severe symptomatic hypercalcemia.

Here we describe a case of SCFN which was complicated by severe hypercalcemia in an infant successfully treated with a single dose of intravenous pamidronate.

## Case Report

A female newborn was delivered in at 40 + 2 weeks of gestation *via* emergency c-section due to absence of fetal movements, non-reassuring fetal status and extreme bradycardia at fetal echocardiography. The mother was a healthy 32-year-old caucasian female with no previously known medical issues. No problems during pregnancy until that moment have been reported. Birth weight of the infant was 4,070 g.

She required intubation and complete cardiopulmonary resuscitation with 3 doses of epinephrine at the delivery. APGAR scores were 0, 3 at 1 and 5 min, respectively. After 17 min the heart rate increased to over 70 beats/min. Passive cooling was initiated.

Arterial blood cord analysis showed severe metabolic acidosis (pH 6.86, base excess: −26 mmol/L) which persisted at 30 min of life (pH 6.51, PCO2 110 mmHg HCO3 8 mEq/L, base excess: −26 mmol/L, lactate 30 mmol/L). She also had hypoglycemia (12 mg/dL) which resolved after a 2 mL/kg bolus of 10% dextrose.

**FIGURE 1:**
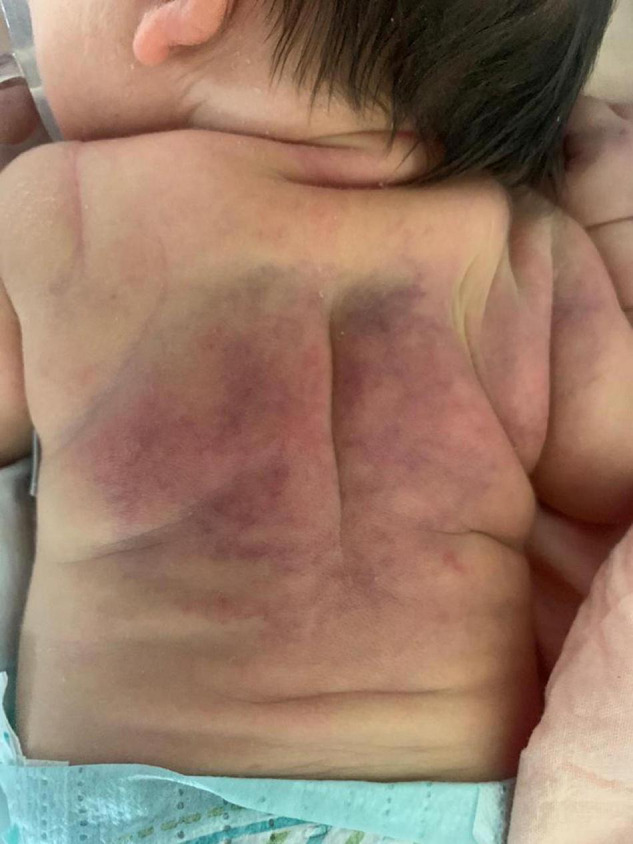
Skin lesion on the back and the shoulder.

**FIGURE 2:**
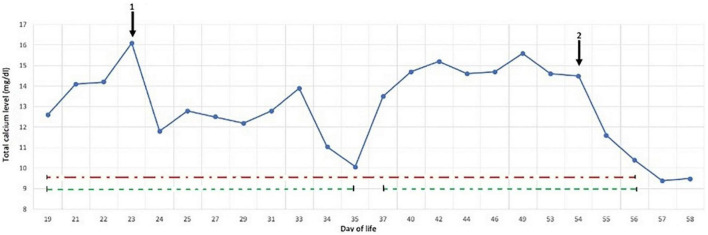
Response of calcium levels to main treatments: hydrocortisone (1) suspendend at day 29, and pamidronate (2), diuretic therapy (dashed green line), hyperhydration (dash/dot red line).

At 4 h of life, the neonate was transferred with mechanical ventilation support to our Neonatal Intensive Care Unit; upon arrival, the electroencephalogram showed hypovolted patterns and therapeutic hypothermia was performed for 72 h. Under cooling, anticonvulsant therapy for seizures and inotropes for hypotension were administered. Three days after rewarming she developed indurated, erythematous large plaques on her back and shoulders (see Figure 1), which were consistent with SCFN which was also confirmed by dermatological consultations. Calcium levels were monitored during the first 2 weeks of life and were within normal limits, however on day 19 her calcium level was 12.6 mg/dL (normal value range 8.5–10.5 mg/dL). The parathyroid hormone (PTH) was suppressed (2 pg/mL; normal 15–65 pg/mL), while 25 hydroxyvitamin D3 was low (24 ng/mL; sufficiency > 30 ng/mL) and the 1,25-dihydroxyvitamin D3 was elevated (125 ng/mL; normal 25–86 ng/mL). This confirmed the diagnosis of hypercalcemia deriving from SCFN (11). As a result, she started hyperhydration (daily fluid intake: formulated milk 150 ml/kg and intravenous saline solution 50 ml/kg) and diuretic therapy with furosemide with 3 mg/kg/die and spironolactone 1 mg/kg/die. Despite the therapy, on day 23 total calcium levels increased to 16.1 mg/dL and Ca^++^ 1.84 mmol/L, so treatment with hydrocortisone (2 mg/kg/die) was initiated and administered for 7 days in graduated doses. On day 29 total calcium levels reduced to 12.2 mg/dL and hydrocortisone was discontinued but furosemide and spironolactone were administered until day 35 when normal calcium levels were finally achieved (10.7 mg/dL). Just 2 days after the interruption of treatment, due to of a rebound in serum calcium levels (13.5 mg/dL), diuretic therapy resumed. On day 54, given the constant level of total calcium around 14.5 mg/dL and Ca^++^ 1.8 mmol/L despite therapy, a single dose of pamidronate at 0.25 mg/kg was given. After the first administration the calcium level rapidly decreased to 11.6 mg/dL in about 24 h and to 10.4 mg/dL the following day. No side effects were reported apart from a single episode of fever recording at 39°C the day after the infusion. On day 57 and day 58 total calcium levels were 9.4 and 9.5 mg/dL, respectively with no additional therapy (Figure 2).

Renal ultrasound was regularly performed during hospitalization, and prior to discharge showed no signs of nephrocalcinosis with normal renal function and normal urinary creatinine and calcium ratio. Electrocardiograms did not show abnormalities in rhythm or electrical changes.

The neonate was successfully discharged in good health, with serum calcium level of 9.5 mg/dL, 25 hydroxyvitamin D3 of 19.1 ng/ml and 1,25-dihydroxyvitamin D3 of 110 ng/mL. No therapy was prescribed. One week post discharge, calcium levels were 10.5 mg/dL and remained stable until the next week. Regular follow-up visits were scheduled with regular monitoring of calcium levels (minimum 10.2 mg/dL; maximum 10.9 mg/dL).

Four months later, the calcium levels were 10.2 mg/dL, 25 hydroxyvitamin D3 was normal (36 ng/mL) as well as PTH value (19 pg/mL) while 1,25-dihydroxyvitamin D3 was 100 ng/mL. Supplementation with oral 25 hydroxyvitamin D3 400UI/die was initiated.

## Discussion

Subcutaneous fat necrosis of the newborn is a transient inflammatory disorder of adipose tissue associated with fetal and neonatal stress with a benign course disappearing within 3–6 months without treatment (1). The incidence of hypercalcemia as a complication seems to be low, and usually appears between 1 and 6 weeks following the onset of skin lesions and can last for months. The etiology of the hypercalcemia is unclear. Recent observations suggests that hypercalcemia is linked to extrarenal expression of 1α-hydroxylase that leads to dysregulated productions of 1,25-dihydroxyvitamin D3 (11).

Within the skin, expression of 1α-hydroxylase is normally restricted to the basal layer of the epidermis, and it is expressed in other cells such as macrophages following immunological challenge (12). This increased expression in granulomatous inflammatory cells results in conversion of 25-hydroxyvitamin D3 to its active form of 1,25-dihydroxyvitamin D3 thus eventually leading to increased intestinal calcium absorption (11–13).

Our patient presented with elevated 1,25-dihydroxyvitamin D3 levels with undetectable PTH levels supporting the hypothesis of unregulated production 1,25-dihydroxyvitamin D3.

With classic treatment regimens for hypercalcemia, the decrease in calcium is slow and treatment does not change the natural course of the disease and its complications. Furthermore, treatment with corticosteroids and furosemide is associated with renal calcium excretion and increased risk of nephrocalcinosis.

The failure of first line therapies for hypercalcemia and the risks of collateral effects of corticosteroids make bisphosphonates a valid alternative. The bisphosphonates attach to hydroxyapatite binding sites on bony surfaces inhibiting osteoclastic bone resorption. When osteoclasts begin to resorb bone that is rich in bisphosphonate, it is unable to adhere to the bony surface, and to continue bone resorption. Bisphosphonates also promotes osteoclast apoptosis decreasing osteoclast progenitor development and recruitment (14). For these reasons they are used to treat infants with osteogenesis imperfecta (15) and results in a decrease in serum calcium. Since they reduce the renal calcium load, it does not increase the risk of nephrocalcinosis. Side effects can include an acute “flu-phase” reaction with fever, myalgia, bone pain, vomiting, and hypocalcemia (15). A destructive side effect is osteonecrosis of the jaw which is mostly seen in adult patients with repeated dose of bisphosphonate (16). Fortunately, a recent systematic review has shown that this complication in pediatric age is not present (17). According to literature, there is not much evidence regarding pamidronate in the neonatal population, however a dosage of 0.25–0.5 mg/kg has been found to be rapidly effective and well tolerated (18, 19).

In our patient, pamidronate was given after the unsuccessful use of the classic treatment regimens. Calcium levels normalized after one single dose of pamidronate 0.25 mg/kg ev in 4 h with minimal use of furosemide. Pamidronate was well tolerated, and a single feverish peak spike was successfully treated with antipyretics. No hypercalcemia was detected during follow-up, and no other therapy or supple doses of pamidronate were necessary unlike what is described in literature so far (18). No nephrocalcinosis was noted on renal ultrasounds.

Alos et al. (18) suggested that pamidronate could be considered as first-line treatment for severe hypercalcemia in SCFN with the aim of reducing nephrocalcinosis. In their report 2 out of 4 patients treated, however, developed nephrocalcinosis.

Lombet et al. (20) reported a case who was successfully treated with 3 doses of pamidronate after first line of IV fluids, diuretics and corticosteroids therapy failed. Steroids and diuretics were then prolonged and tapered for 1 months. Khan et al. (21) described a 7 weeks old treated with a similar therapeutic protocol. Also these cases presented nephrocalcinosis. A possible explanation given was the degree and the duration of hypercalcemia.

Our case, despite a Ca++ higher than the suggested threshold Ca++ level of 1.75 mmol/l, did not present nephrocalcinosis. Moreover, we discontinued the diuretic therapy and the steroids sooner than reported in literature. Probably this contributed to reduce renal calcium excretion. Last but not least, our patient did not need supplemental doses of pamidronate.

## Conclusion

Subcutaneous fat necrosis is a rare complication of neonatal stress, in particular of therapeutic hypothermia, and it may evolve into a life-threatening condition if complicated by severe hypercalcemia. In order to prevent complications of hypercalcemia in SCFN, it is important to identify hypercalcemia early. When high levels of calcium do not respond to hyperhydration and diuretic therapy, physicians should think about the use of bisphosphonates to treat hypercalcemia and to avoid corticosteroids. In our experience, one single dose of pamidronate 0.25 mg/kg has been proven to be rapidly effective and well-tolerated in the short-term.

Further studies are needed to understand the pathophysiology of SCFN, on the impact of pamidronate and other bisphosphonates on the natural history of SCFN and to confirm the safety of this treatment in neonatal patients. Moreover, screening for hypercalcemia is recommended given the risk of this complication at different ages and the natural history of the subcutaneous nodules.

## Data Availability Statement

The raw data supporting the conclusions of this article will be made available by the authors, without undue reservation.

## Ethics Statement

Ethical review and approval was not required for the study on human participants in accordance with the local legislation and institutional requirements. Written informed consent to participate in this study was provided by the participants’ legal guardian/next of kin. Written informed consent was obtained from the minor(s)’ legal guardian/next of kin for the publication of any potentially identifiable images or data included in this article.

## Author Contributions

SM coordinated the clinical decisions on the patient, conceptualized and designed the study, supervised and helped data collection, drafted the final version of the manuscript, and critically reviewed it for important intellectual content. MP, IG, and OV collected the data and drafted the initial manuscript and reviewed it. TR and GB helped in the clinical management of the case, designed the data collection instruments, and critically reviewed the manuscript. All authors approved the final manuscript as submitted and agreed to be accountable for all aspects of the work.

## Conflict of Interest

The authors declare that the research was conducted in the absence of any commercial or financial relationships that could be construed as a potential conflict of interest.

## Publisher’s Note

All claims expressed in this article are solely those of the authors and do not necessarily represent those of their affiliated organizations, or those of the publisher, the editors and the reviewers. Any product that may be evaluated in this article, or claim that may be made by its manufacturer, is not guaranteed or endorsed by the publisher.
